# A qualitative study to understand the facilitators of and barriers to retention in care to the national PMTCT Option B+ programme in Uganda

**DOI:** 10.1371/journal.pone.0314885

**Published:** 2025-01-16

**Authors:** Charity Kyomugisha-Nuwagaba, Rachel King, Rose Baryamutuma, Simon Muhumuza, Linda N. Kisakye, William Bazeyo, Evelyn Akello

**Affiliations:** 1 Monitoring and Evaluation Technical Support, Makerere University School of Public Health, Kampala, Uganda; 2 Institute for Global Health Sciences, University of California, San Francisco, San Francisco, California, United States of America; 3 Ministry of Health, AIDS Control Program, Kampala, Uganda; University of Zimbabwe Faculty of Medicine: University of Zimbabwe College of Health Sciences, ZIMBABWE

## Abstract

**Introduction:**

Effective prevention of mother to child transmission (PMTCT) programmes require women and their infants to have access to a cascade of HIV care and treatment interventions. Retention in care reduces the risk of vertical transmission and opportunistic infections among mothers living with HIV. Uganda has made great strides in ensuring the success of the prevention of mother to child transmission program. Although an increasing number of people living with HIV (PLHIV) in Uganda are benefiting from the rapid scale-up of antiretroviral therapy (ART), retention in HIV care and treatment services remains a major concern. Identifying and understanding the reasons for dropping out of care among mothers enrolled in the Option B+ program among those who were retained and those who dropped out is key to inform policy and program practice.

**Methods:**

We conducted a qualitative study to understand the facilitators of retention and reasons for loss to follow-up among HIV positive mothers in central Uganda who engaged in the Option B+ program. We conducted 29 focus group discussions (FGDs) with Village Health Teams (VHT) and ‘Peer Mothers’. We performed 21 in depth interviews (IDI) with mothers who had been lost to follow up during the post-partum period, and 27 among those who remained in care. These were conducted in 18 districts in Central Uganda.

**Results:**

Participants identified barriers and facilitators to retention in HIV care. Barriers included self-stigma and fear of disclosure, mental health challenges, community perceptions, poor health provider attitudes and structural challenges, lack of transportation and food, long waiting time at health facilities and client mobility. Both the clients retained and not retained in care discussed mental illness, feeling sick and competing priorities as barriers. Facilitators for retention in care included adequate community support systems, early initiation on ART, giving birth to HIV negative children and economic stability. These were noted as key enabling factors for retention. It was also highlighted that presence of friendly clinic staff members, scheduling reminders were important aspects of retention.

**Conclusion:**

Findings highlighting barriers covering personal, interpersonal, structural and community suggest that developing client-centered models addressing social and community barriers and provide more holistic services is key to retaining mother-infant pairs in care. Emphasis on the use of community health workers and provision of financing, as well as institutionalization of quality improvement would provide alternatives for overcoming barriers to retention in care.

## Introduction

It is widely recognized, globally, that attrition from the prevention of mother-to-child HIV transmission (PMTCT) cascade is a significant obstacle to achieving UNAIDS’ and the World Health Organization (WHO)’s goal to eliminate mother-to-child transmission [1–5]. Transmission of HIV from an infected mother to her child during pregnancy, childbirth, or breastfeeding (MTCT), remains one of the most frequent modes of HIV infection in sub-Saharan Africa [6]. The risk of HIV transmission from mother to child can be as high as 15–30% during pregnancy and 10–20% during breastfeeding if treatment for the mother and prophylaxis for the exposed infant are not provided [7, 8]. With recommendations for early HIV testing of pregnant mothers and antiretroviral treatment the risk reduces to less than 5% in the breastfeeding population and to less than 2% in the non-breastfeeding population [7, 9].

It is estimated that about 25,000 babies would be born in Uganda with HIV every year if no intervention were instituted and without treatment, half of the children living with HIV would die before their second birthday [2, 10]. Thus, the current PMTCT program, as reported in a recent impact evaluation, is a highly effective intervention and has significant potential to improve maternal and child health and survival [6]. Option B+ as an approach has been widely accepted as a national program since its inception but has been shown to have differing outcomes with different sub-populations [11–13]. For example, younger mothers, 15–24 years old have a higher transmission rate than their older counterparts [9]. The Option B+ WHO and national guidelines that stipulate all pregnant women start on triple antiretrovirals irrespective of CD4 cell count and continue for life and infants are given daily nevirapine or zidovudine from birth to 4–6 weeks [14].

Uganda signed the Global Plan towards virtual elimination of new HIV infections among children. As part of this plan, the country embarked on Option B plus (Option B+) based on the decision to start ART at any stage of a woman’s HIV infection. Lifelong ART (Option B+) is currently the global and national recommended treatment strategy and is implemented in all districts in Uganda.

For optimal clinical outcomes in individuals on ART to be realized, retention in care is required. The definition of retention in care is highly debated in current literature [15–17]. For this study, retained in care was defined as “those known to be alive and receiving ART at the end of a follow-up period or 18 months post-initiation of ART [18]. Failure to remain in care is often associated with cessation of medication, clinical deterioration and high risk of death [15]. Several studies conducted in Uganda have documented suboptimal retention in care [19]. Long distances to health facilities, financial constraints, food insecurity, ART toxicities, stigma, inadequate social and partner support, lack of disclosure and belief in divine healing have been reported as some of the major factors underpinning retention failure [20–23].

There continue to be gaps in the national PMTCT Option B+ program, especially with regards to retention in care [9]. Understanding the reasons for loss to follow-up is critical to inform current implementation and to inform policy and program improvements [24]. With this in mind, we conducted a comprehensive evaluation in 24 districts of Uganda to measure the effectiveness of Option B+ strategy on the MTCT rate and on survival of mothers and infants up to 18 months of age [25]. As part of this evaluation, a qualitative component was undertaken in 18 of the 24 districts that aimed at identifying and understanding the reasons for dropping out of care among mothers enrolled in the Option B+ program among those who were retained and those who dropped out to inform policy and program practice.

## Methods

We conducted a cross sectional qualitative descriptive study to understand the facilitators of retention and reasons for loss to follow up among HIV infected mothers in central Uganda who engaged in the Option B+ program [26]. We conducted 29 focus group discussions (FGDs) with Village Health Teams (VHT) and ‘Peer Mothers’ between 1 March-30 April 2015. We also performed 21 in depth interviews (IDI) with mothers who had been lost to follow up during the post-partum period, and 27 among those who were still retained in care during the study period. All these were conducted in 18 districts in Central, Uganda.

The VHTs are trained community health workers and are recognized in the healthcare system as the first level of health service providers. Their roles in the implementation of the Option B+ program include but are not limited to; identification and linkage of pregnant women to health facilities, follow-up and sometimes basic primary health care. The peer mothers are HIV positive clients who have gone through the PMTCT program. Their role in the PMTCT program is normally psychosocial support through sharing of personal experiences with the pregnant and lactating HIV positive mothers. Their activities are expected to strengthen retention in care. They also provide support to health workers by performing a wide range of activities such as file retrieval, health education, follow-up of mothers in communities and counselling and sharing of their own experiences.

### Sampling and recruitment

In the central region of Uganda where the evaluation was conducted, within the 24 districts, the facility in each district with the lowest retention rate, based on the District Health Officer and PMTCT focal persons’ assessment was identified. Women currently or previously in PMTCT were identified from 18 of the 24 facilities. Qualitative interviews were conducted at 10 hospitals, 11 Health Centre IVs and 18 HC IIIs. At each facility, the person in charge of the administration of the facility, contacted the VHT members and peer mothers affiliated to that site and invited them for the FGDs.

Qualitative interviews were conducted among respondents drawn from hospitals, Health Centre IVs, and Health Centre IIIs.

Participants in focus group discussions comprised of members who have worked with the PMTCT programme for at least six months. The number of respondents who participated in FGDs ranged from 6–10. In-depth Interviews (IDIs) were conducted with HIV-positive mothers retained in care. These comprised of mothers retained in care during pregnancy and post-partum period and could be traced by health care workers and volunteers. IDIs with mothers lost to follow up (LTFU) who comprised of HIV-positive or breastfeeding mothers initiated on ART who dropped out care for at least three months, could be traced and were willing to participate in the study.

Mothers retained in care were consecutively selected from the list of mothers whose records had been abstracted for the quantitative study. These comprised of mothers who had been retained for 18 months. Mothers LTFU were purposively selected by the VHT and facility staff from a list of those who were not retained in care. Nearly all the women on the lists of lost to follow up were contacted by the VHTs on their mobile phones. For mothers who could not be reached by phone, the VHT member or peer mother went to their home and explained the objectives and methods of the study and asked to meet at a place of their convenience for the interviews. Participants who declined to be part of this study were replaced.

### Data collection

Interview guides were used during FGD and IDI to enable a detailed exploration of both mothers’ and health workers’ views of and experiences with the PMTCT program. Most interviews were conducted in Luganda and lasted between 45 minutes to one hour, and were moderated by 2 project staff, one who directed the conversation and the other who took notes; interviews and FGD were audio recorded with permission from the participants. The topic guide included the following prompts to elicit participants views and experiences: i) understanding of the PMTCT programme; ii) experience with following up mothers and community response to the program; iii) perceptions of the quality of care mothers receive at the facility; iv) challenges and perceived as well as barriers towards the option B+ program; v) facilitators of retention and reasons for loss to follow up and suggestions for improving the program.

### Data processing and analysis

Audio recordings were transcribed from either the English audio or the Luganda audio directly into English and were analyzed by three different project investigators who identified themes and developed codes and their definitions. Divergent views were resolved through discussion. In addition, transcripts and notes were analysed using NVivo version 10 (QSR International Pty. Ltd, Victoria, Australia). Quotes were selected based on how illustrative they were of major themes or sub themes. The thematic areas and the quotes supporting them, were then analysed further to explain the themes in more depth.

### Ethical review and informed consent

Ethical clearance was obtained from Makerere University School of Public Health Research and Ethics Committee and approved by the Uganda National Council of Science and Technology. The protocol was also reviewed and approved by the US Centers for Disease Control Office of the Associate Director of Science. The study was explained to participants in the local language followed by signed informed consent. All participants were reimbursed with 18,000 Ugx (approximately 5 USD) for their participation.

## Results

The results are presented in three thematic areas emerging from the data namely, main benefits of the PMTCT programme, barriers to retention and facilitators of retention.

### Main benefits of PMTCT programme; structural factors

Effective PMTCT programmes require women and infants to receive a cascade of interventions ranging from antenatal services, HIV testing during pregnancy, ART, safe birth practices, appropriate infant feeding and other postnatal health care services. Overall, HIV positive mothers identified the following benefits from the PMTCT programme: knowledge about HIV and the PMTCT programme, access to ARVs with improved health, the receiving counselling about care and availability of support systems through the community health system, and the potential for having an HIV negative child.

#### Knowledge about HIV and the PMTCT programme

Awareness and knowledge regarding the PMTCT programme is important for informed decision making and contributes to the decision to remain in care. Mothers indicated that the knowledge gained from the programme helped them make decisions around enrolling in ART, disclosure and most importantly for positive living and retention in care.

“*We learnt so much from the PMTCT programme*. *The health education about this programme has given us information on how to take care of ourselves and the dangers of not being on treatment if one is HIV positive*. *This has enabled us to stay in care and also educate fellow mothers who are struggling with HIV”*

(Mother retained in care, Mityana District).

#### Access to ARVs with improved health

Adherence to ART is one of the most important contributing factors to positive clinical outcomes in patients with HIV. For both mothers retained in care and those lost to follow up, being able to access ARVs was identified as one of the major benefits from the PMTCT programme. Participants reported that unlike other medicines, ARVs are readily available and the fact that they do not need to pay for them made this access even more important.

“*The greatest benefit is being able to access ARVs*. *I can imagine a situation where I am sick and I have to buy these medicines*.*” “ARVs! We can get ARVs every time we come to the facility and that is very important”*

(Mother retained in care, Gomba District).

Access to ARVs was reported to be important for improved health:

*“Drugs (ARVs) have helped me a lot because I used to have a lot of discomfort in my nails as if they were on fire and I didn’t know the reason why*. *I had even started getting itchy black spots on my skin but ever since I started this medication they stopped; I feel very healthy and even when I fall sick I come to the health centre and access medicine*. *These drugs have helped me a lot”*

(Mother Retained in Care, Nakaseke District)

### Availability of counselling services

The formation of active support groups is an important factor for implementing PMTCT programs as a community network of people living with HIV is able to identify HIV positive clients in need and support them to enroll and stay in care. Some of the major benefits identified were counselling services and support groups.

“*If it was not for the counselling received in the support group, I would not be here*. *I am comforted by fellow mothers and can share challenges with people who understand what I am going through*.*”*

(Mother retained in care, Mubende District.)

“*We receive counselling*. *Counselling is the best benefit*. *Without it*, *we would die*”

(Mother retained in care, Luwero District)

### Barriers to retention

Several barriers were identified as the reasons for loss to follow up. Overall, key individual factors included fear of disclosure and stigma, as well as difficulty with the medication related to drug side effects. Other barriers included negative community perceptions about HIV drugs, negative experiences with waiting time, and poor health provider attitudes. Some mothers who were lost to follow up simply claimed they were reluctant to come back to the clinic without citing specific reasons. Lack of food and transportation also prevented some women from adhering to the program.

#### Violence, stigma and disclosure

Lack of disclosure and self-stigma played significant roles in adherence to the PMTCT program. Fear of disclosing was often interwoven with gender and power issues in the home and community. Many participants reported hesitating to disclose their status to family, friends, and acquaintances. Uncertainty about how family, friends, or the public would respond to their status made some patients anxious and affected their ability to attend appointments. Peer mothers described the potential positive and negative consequences of HIV sero-status disclosure. Mothers who were not retained in care also mentioned that they did not want their husbands or other family members to know that they were HIV positive. Among fishing communities, both on the islands and along the lake shore, women mentioned that fear of stigma was particularly high among first time young mothers and that some mothers were afraid of ART.

“*After women have received their results, we often ask them whom they will disclose this information to and most times they say that they will not talk to anyone about it and this means that if this woman is married, she will not tell the husband and mostly likely she will not come for treatment”*

(Peer Mother, Lyantonde District*)*.

*[with respect to disclosure] “Some people fear their husbands while others fear community members like neighbors*.

(Peer Mother, Luweero District).

Stigma has far reaching consequences, both men and women fear being identified in the clinic so will avoid coming for HIV testing and to collect their drugs.

“*Some people gossip a lot about their friends who are HIV positive and on treatment and this increases the stigma in the community which leads to dropping out of the program*.

(FGD peer mothers, Kiboga District).

Disclosure of HIV status was difficult for many mothers. All groups interviewed i.e. mothers lost to follow-up, mothers retained in care, peer mothers and Village Health Teams (VHTs) discussed the difficulties women faced when they were given their HIV positive result when they did not know the HIV status of their husband or partner. Some mentioned that when women disclosed to their husbands, this led to domestic violence, separation and in some cases, husbands brought home second wives.

“*When* s*ome pregnant women test positive and are given ARVs, they throw them away on the way back home, saying that they didn’t want their husbands to send them away after knowing their status*.*”*

(Mother, Retained in Care, Mukono District).

*“Most women who abandoned the program have challenges in their homes*. *Many undergo domestic violence*. *Some missed coming back on the appointment dates because of such challenges like [fear of] divorce*. *The next time they realize that they are pregnant*, *and they do not even come back to deliver in hospital to save the child*.

*(*Village Health Team, Masaka District).

#### Mental illness, stress and other drug side effects

Participants identified symptoms of mental illness such as stress, anxiety, and nightmares as barriers to remaining in care. Participants who reported feeling depressed described sometimes not wanting to “bother” with traveling to the clinic. In addition, participants felt apathetic about their health care, with some stating that they did not care whether they lived or died. In addition, feeling sick was a barrier and was commonly a reason for skipping or rescheduling appointments. This was also often related to side effects of ARVs such as dizziness, nausea, vomiting, and weakness, as the reasons they dropped out of care.

*“I found challenges with the medicine*. *I used to feel dizziness only that health workers told us that we are likely to get bad dreams, feeling like you are drunkard- I got scared”*

*(*Mother, lost to follow up, Mukono)

*“I used to take ARVs and I found out that I was getting worse*. *Whenever I took the drugs*, *I would run mad*, *I can be like a crazy person*. *Whenever I would take it*, *I got mental instability*, *I got uncoordinated speech and they had to tie me up like a mad person*. *I used to be like a person who has taken alcohol*, *as if I am drunk”*

(Mother, Lost to Follow Up, Lyantonde District).

#### Negative community perceptions of HIV drugs

Community views, norms and perceptions can have an impact on whether mothers will continue with the PMTCT programme. A woman described a community perception regarding HIV drugs:

“*I heard that people who take HIV drugs get mental problems*. *For example, we have a man in the community who ran mad [mental illness] and people said that it was as a result of taking HIV drugs and I think this scares people from testing for HIV*

(Mother Retained in Care Lyantonde District).

Another peer mother described slightly different community views of ART: “*Some people fear to take ARVs because there are some people who die suddenly*, *and the community members say it is because of the ARVs they have been taking*.*”* (Peer mother Luweero District).

#### Health provider attitudes

Having positive relationships with health care providers increased confidence in the provider’s recommendations and advice. Moreover, having a supportive patient-provider relationship creates a sense of trust, allowing patients to honestly share their health experiences and adherence behaviours. However, poor health provider attitudes were identified as barriers to retention in care. This, coupled with long waiting time in the facility discouraged patients from attending. Participants reported that mothers often preferred to stay away and/or use the services of traditional birth attendants where they were sure to find kind and supportive care. Participants were also unwilling to attend clinics if they felt disrespected and /or patronized.

“*After giving birth I went back to the hospital and the health worker I found there was so abusive*. *She was telling me that I was stupid*. *But I had heard from a friend who was also HIV positive that when you are sick and you are breastfeeding, you should go to the hospital when you feel some discomfort in the breast*. *But for me when I went, I was told that I should breast feed until the child is one year old, and me I thought I was supposed to stop at 6 months*. *So, the nurse became so abusive and told me to follow my own knowledge*. *I never went back*.

(Mother, lost to follow up Mpigi District).

“*Some health workers have a bad approach; they are rude and inconsiderate*. *When people get to know that they are [HIV] positive they get stressed and think the world is ending for them*. *If they [health workers] speak to you rudely*, *you get discouraged and you may just decide to give up attending care and wait to die*. *It’s hard for one to come back”*

(Peer mother Luweero District).

#### Cost of transportation and food

The cost of transportation for monthly clinic visits was identified as a reason for loss to follow-up. Almost all respondents lost to follow up cited the need for transport funds as a source of anxiety and a key factor for missed doses and clinic appointments. Participants reported that they struggled with finding money for transport and other necessities such as food and family welfare. Findings suggest that transport costs can compromise adherence, access to and retention in care.

*“Since I stay far away from the health facility, it becomes costly for me to access care since sometimes I have no money for transport and I cannot ask my husband since I have never disclosed [my HIV status] to him*.

(Mother lost to follow up, Masaka District)

Lack of food or hunger was cited as a reason for loss to follow-up. Both mothers retained and not retained reported that insufficient food was a challenge. Insufficient food was mostly related to poverty and participants indicated that the poorer households found it more difficult to take ARVs since it was a requirement that they eat well while on treatment.

#### Negative experiences with waiting time

Both the retained and non-retained clients referenced negative experiences at their clinic. Several participants mentioned frustration about clinic wait times that often extended to several hours. Many participants disliked the waiting room experience for a range of reasons including fear of unintentional disclosures. Challenges with long waits for laboratory testing were also mentioned as barriers to appointment adherence, and eventually loss to follow up.

“*We take too long at the clinic sometimes and our work at home suffers*. *The health workers sometimes come late and then the lines become too long*. *Even when we are waiting, it is possible for people whom you don’t want to know that you are positive may come and see you in the ART clinic and they start talking to the whole village about you*.*”*

(Mother Lost to Follow Up, Nakaseke District)

#### Client mobility

Client mobility affects retention in care for mothers as well as infants (i.e. whose caregivers might relocate seasonally to seek work. Participants indicated that migration was one of the reasons for undocumented transfer or loss to follow up. Migration cases were either due to work or in some cases young women migrated for a new relationship and a new life.

*The main challenge we are facing is that, being near the landing site, we enroll mothers very well but after say only two visits, you will not see them again*. *Others shift to landing sites where fish business is booming at that time and by the time they return, you find when they have already given birth and have left the drugs where she delivered from; others do not come back at all”*.

(VHT member, Mukono District)

### Facilitators of retention in care; community, family and personal levels

Retention in care is necessary for ongoing access to ART, to identify cases of treatment failure and to monitor for any drug toxicity. It also provides additional benefits of social support, and secondary prevention messages that can help patients navigate a lifelong treatment. Facilitators of retention reported included social support, ability to start ART early, having an HIV-negative child, and financial stability.

#### Positive social support systems

For clients, the positive relationships with Village Health Teams, peer mothers and clinic staff were commonly discussed facilitators for remaining in care. Patients reported that a strong relationship with their support system increased confidence in their provider’s advice. Moreover, having a supportive patient-provider and spouse related relationship created a sense of trust, allowing patients to honestly share their health experiences and adherence behaviours. Both the clients retained and non-retained commonly referenced people in their lives who supported their appointment adherence. Husbands, siblings and support groups were all discussed as important sources of support. These people reminded and motivated participants to attend appointments. Mothers reported that social relationships with husbands, peer mothers and follow up from VHTs helped to overcome barriers to care.

*When I went back home and told him [husband] that I tested HIV positive, he said “that is very good*. *I am also HIV positive but I feared to tell you because when some men reveal their status to their wives, they decide to separate or divorce and leave the children behind*. *Now that you have known your status, you can start taking HIV drugs from here because for me I take my drugs from Mulago hospital”*. *I like the fact that I was able to tell him because whenever he comes home, we keep on reminding each other whenever it is time to take our drugs”*.

(Mother retained in care, Mubende District)

*“My husband is negative*, *and I am positive but he allows me to take ART drugs*. *He is the one who reminds me to take the drugs and also to give to the children”*

(Mother retained in care In-depth Interview, Mityana District).

#### Starting treatment early

Mothers retained in care often talked about starting treatment early as one of the facilitators for retention in care. They reported that they felt healthier and stronger on treatment, felt improvements in their mental health because of their hope for a healthier life, experienced less stigma because people cannot tell they are on treatment, and that they were more encouraged to attend health centres because there is certainty that treatment is available to them. They also attributed ability to get treatment early as having saved them from recurrent infections, promoting healthier lifestyles and enabling access to information for positive living.

“*The other thing that I have realized is that when you start treatment early enough, you don’t get any complications or side effects and the body is always healthy and energetic but when you start the treatment late, many issues might arise that will lead to everyone identifying you as HIV positive*”

(Mother Retained in care, Lyantonde District.)

#### HIV negative children

The desire to prevent vertical transmission was a frequently mentioned motivator for uptake, retention and adherence to PMTCT care. Both retained and non-retained participants reported that the desire and realization that they could have HIV negative children was key.

*“I really desired to have a healthy baby and I managed to achieve this and so I have to strive a lot to make sure that I don’t infect him now”*

*(*Mother retained in care, Wakiso District)

## Discussion

Retention in care is critical to prevent vertical transmission of HIV. Our study findings contribute to the current literature by examining the barriers to and facilitators of retention in a study among participants who were retained and those who dropped out as well as peers and health care providers. Our results show that pregnant women have some unique challenges with retention in care that need to be addressed. Pregnant and lactating mothers had challenges with stigma and disclosure, poor health provider attitudes, long waiting time, inadequate food, transportation to health facilities, stress and mental health challenges with related side effects.

Overall, the study participants demonstrated high knowledge of Option B+ and related services. Despite the awareness and knowledge of the importance of the programme, many women still struggled with disclosing their status to family and friends due to stigma. Non-retained individuals commonly cited stigma as a barrier to remaining in care compared to retained individuals. This difference may be a consequence of different perceptions and experiences in the community and at health facilities. Patients retained in care may also have had stronger social support systems or access to mental health care, which have been identified as protective against stigma than those not retained in care [27]. Additional studies would be important to provide a clearer understanding on how clients perceive stigma and what impact it may have on health behaviour. It is clear that stigma is a significant challenge and may therefore undermine the efficacy of Option B+, because mothers not coming into facilities for treatment further affects the health of women with higher likelihood of transmission.

Poor health provider attitudes also hindered retention in care. As in other studies, we found that structural factors that included poor quality of pre-/post-test counselling, the lack of adequate, motivated and skilled manpower, high staff turnover, and poor quality of service delivery have contributed to loss to follow-up in HIV programmes [23, 28]. In addition, poor or no monitoring of referral systems for HIV positive women meant it was difficult to find those who were lost to follow-up even if they go to other facilities to give birth. It is therefore important to reinforce and ensure referral structures to enable follow up and support existing services. This will require investments in cost effective administrative and information sharing facilities at all levels, while ensuring confidentiality and privacy of clients.

This study indicates that inability to stay in care was further complicated by cost of accessing services. Low incomes meant many women could not afford transportation and other requirements for accessing PMTCT services that were offered for free. This is even more challenging for rural populations who find transportation cost to the clinics expensive due to the long distances and generally low incomes in this population. This means that even when women recognize and are convinced about the importance to access care, transportation costs may inhibit access to services. Differences in income and access to individuals who can provide transportation may explain this finding. Some studies have indicated that clinics providing support services, including transportation and case management, have better retention rates than those without these services [29]. However, this may not be a sustainable approach in this context.

Negative experiences with long waiting time and poor health provider attitudes were noted among those retained in care and those lost to follow up. Similar to other studies, satisfaction with the health provider experience was an important factor that influenced whether or not patients returned for care [23, 30]. In addition, clients’ experiences at health facilities including waiting time depends on the quality of care but also on interactions and relationships with health providers.

Participants described difficulties with continuously attending health facility visits, including dealing with competing priorities, mental illness, stress and lacking finances. It is worth noting that these people, particularly in low-income populations, may experience social and economic strains due to no or little income and depressive symptoms. However, both the retained and lost to follow up participants commonly mentioned that positive and supportive patient-provider relationships, better incomes, early initiation on ART and community support were important factors for retention in care and these factors mediated the barriers to attending follow-up visits. Studies on patient and health provider relationships have also indicated that positive health provider attitudes improve patient’s understanding of health issues and commitment to the service provided and eventually lead to better retention in HIV care [29].

Satisfaction with care has been shown to be positively associated with retention in care and adherence to ART [31]. Non-retained patients may be unhappy with their health facility and for that reason do not stay in care. It is vital to conduct more research to gain a better understanding of the differences between patients retained and those lost to follow up even when they have the same demographic characteristics and differ in their explanations about barriers to care.

### Limitations

The study did not collect information on the demographic characteristics of the participants, therefore we were not able to understand the barriers and facilitators across the different demographic categories such as age. We also note that there could have been reporting bias in the findings and participant responses may have been influenced by social desirability. However, ensuring confidentiality and training interviewers to avoid judgmental questions and reactions helped minimize this risk. Finally, the findings of this study may not be generalized to other populations, as our patients, clinical practices, and geographic and cultural environment may differ from others. Moreover, not all barriers and facilitators identified may apply to the same degree across populations.

### Conclusions

Overall, tailored interventions to ensure mitigation of the impact of HIV require consistent and dedicated involvement of many stakeholders including multiple layers of the health system including policy makers, health providers, families, and communities [4]. Enforcement of guidelines regarding how health care workers treat patients; monitoring of actual follow-up rates and contacting clients for return visits need to be supported further. Emphasis on the use of community health workers and provision of financing, as well as institutionalization of quality improvement would provide alternatives for overcoming barriers to retention in care.
